# Effect of phosphodiesterase inhibitors on platelet function

**DOI:** 10.1016/j.bbrep.2025.102115

**Published:** 2025-06-26

**Authors:** Ravi Hochuli, Valerie Dicenta, Zoi Laspa, Manuel Sigle, Tobias Harm, Tatsiana Castor, Anne-Katrin Rohlfing, Meinrad Paul Gawaz

**Affiliations:** Department of Cardiology and Angiology, University Hospital Tübingen, Eberhard Karls Universität Tübingen, Tübingen, Germany

**Keywords:** Platelets, Phosphodiesterase, PDE inhibitors

## Abstract

Phosphodiesterase enzymes (PDEs) play a pivotal role in regulating platelet activity by modulating intracellular levels of cAMP and cGMP. Modulation of PDE-2, -3 and -5 activity by suitable inhibitors has been found to reduce platelet activity, and thus thrombus formation.

Our aim was to study Ibudilast effects on platelet activation, degranulation and aggregation. Therefore, we used the nonspecific PDE inhibitors IBMX as well as the PDE-5 inhibitor Sildenafil as controls. Platelet agonists collagen-related peptide (CRP-A), adenosine diphosphate (ADP) and thrombin receptor activator peptide (TRAP6) were used to induce distinct activation pathways. PDE inhibition was quantified by western blot analysis. Platelet activity was assessed using flow cytometry, light transmission aggregometry and in vitro thrombus formation.

Inhibition of all platelet PDEs by IBMX substantially reduced platelet activation and aggregation in response to all tested platelet agonists. Ibudilast preferentially inhibits PDE-3 in platelets. Ibudilast decreased platelet activation and aggregation induced by ADP and TRAP, but not CRP-A. Sildenafil alone induced no reduction in PDE activity, platelet activation or aggregation. However, the combination of Sildenafil and Ibudilast had an additive effect on platelet activation. Interestingly, all tested PDE inhibitors demonstrated a significant effect on platelet-dependent thrombus formation.

In conclusion, the effect of PDE inhibitors on platelet function is influenced by two primary factors: the pharmacological target of the inhibitor and the cAMP/cGMP interaction with the activation pathways induced. Platelet activation by ADP via P_2_Y_12_ and TRAP via PAR1 showed a greater response to PDE inhibitors than platelet activation by CRP via GPVI.

## Highlights

• All three PDE isoforms expressed in platelets are involved in regulating platelet function.
• The effect of PDEs on platelet function depends on distinct activation pathways.
• The influence of PDE inhibitors on platelet function is depending on their inhibitory profile.

## Graphical abstract

Graphical illustration of the PDE isoenzymes in platelets and their role in cAMP/cGMP metabolism (**left side**). Influence of PDE activity on platelet activation (**right side**). (ATP, AMP, cAMP: adenosine triphosphate, adenosine monophosphate, cyclic adenosine monophosphate; GTP, GMP, cGMP: guanosine triphosphate, guanosine monophosphate, cyclic guanosine monophosphate; AC: adenylate cyclase; sGC: soluble guanylate cyclase; PKA, PKG: protein kinase A, protein kinase G; NO: nitric oxide; VASP: vasodilator-stimulated phosphoprotein; VASP-P: phosphorylated VASP.

**Figure:**
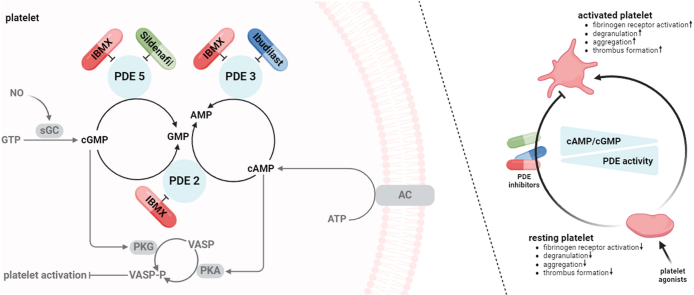

## 1 Introduction

Platelets are circulating corpuscular blood components that play a critical role in thrombosis and hemostasis [1,2]. At site of vascular injury platelets adhere, become activated, aggregate and promote thrombosis [1,3]. Platelet-dependent thrombosis is tightly regulated by plasma membrane surface adhesion receptors [4]. Platelet-dependent arterial thrombosis can be inhibited by blockade of various membrane receptors that communicate with intracellular signaling pathways [5]. Examples for these receptors are the adhesion receptors glycoprotein VI (GPVI) which is activated by collagen binding or the fibrinogen receptor glycoprotein IIb-IIIa (GPIIb-IIIa). Furthermore, G-protein-coupled seven-transmembrane receptors such as adenosine diphosphate (ADP) binding receptor P_2_Y_12_ or the thrombin activated protease-activated receptor 1 (PAR1). Most of these signaling pathways converge in the phospholipase C mediated activation of the protein kinase C and the release of calcium from the intracellular calcium stores.

Cyclic adenosine 3′,5′-monophosphate (cAMP) and cyclic guanosine 3′,5′-monophosphate (cGMP) are intracellular second messengers that are involved in regulation of platelet activation [6,7]. cAMP and cGMP activate protein kinase A (PKA) or protein kinase G (PKG) respectively. Both PKA and PKG have been demonstrated to modulated platelet activation, e.g. by inhibition of the AKT signaling pathway, inositol-3-phosphate (Ins3P) signaling and calcium release from intracellular stores [8]. Cytoplasmic levels of cGMP and cAMP are regulated through phosphodiesterases (PDEs), which catalyze the hydrolysis and thus degradation of both compounds into inactive GMP and AMP [9]. PDEs are key regulators of intracellular cyclic nucleotide levels. The respective isoenzymes of the PDE family (PDE-1-11) exhibit varying affinities for their substrates cAMP and cGMP. PDE-5, -6, and -9 exhibit a preference for the degradation of cGMP, PDE-4, -7 and -8 primarily degrade cAMP and PDE-1-3, −10 and −11 have similar affinities for both cyclic nucleotides [10]. In platelets, the three PDEs PDE-2, -3, and -5 have been described [[9], [11]]. PDE-2 degrades both cAMP and cGMP, whereas PDE-5 hydrolysis cGMP to GMP and PDE-3 cAMP to AMP. Inhibitors of PDE-2 and -3 have been shown to limit hydrolysis of cAMP in platelets, whereas cGMP levels are primarily modulated through PDE-2 and -5 inhibitors [12].

In this study, we applied Ibudilast, a non-selective inhibitor of several PDEs with preference towards PDE-3, PDE-4, PDE-10 and PDE-11 [13], 3-isobutyl-1-methylxanthine (IBMX), a nonspecific PDE inhibitor [14] as well as the potent selective PDE-5 inhibitor Sildenafil [7].

The purpose of this study was to characterize the effect of the three inhibitors with different PDE specificity on platelet function, to disclose the differential effects of PDE-2, 3 and 5 for platelet-mediated thrombosis. We found that all tested PDE inhibitors have a different antiplatelet inhibitory potency depending also on the functional test applied. The most prominent antiplatelet activity was found for the non-selective inhibitor. This indicates that all PDE isoforms known to be expressed in platelets and to regulate both cAMP and cGMP are required during platelet activation and thrombus formation.

## 2 Material and methods

### 2.1 Reagents

Ibudilast was purchased from MedChemExpress (Monmouth Junction, NJ, USA). IBMX (3-isobutyl-1-methylxanthine) and Sildenafil were purchased from Sigma Aldrich Co (St. Louis, MO, USA). Ibudilast and IBMX were dissolved in 100 % dimethylsulfoxide (DMSO; Sigma-Aldrich, St. Louis, MO, USA). Sildenafil was dissolved in 100 % methanol (VWR Chemicals; Darmstadt, Germany). Collagen-related peptide (CRP-A) was purchased from Pplus Products (Salisbury, Wiltshire, UK). Adenosine diphosphate (ADP) was purchased form Chrono-Log Corporation (Havertown, PA, USA). Thrombin Receptor Activating Peptide (TRAP6) was purchased form F. Hoffmann La Roche AG (Basel, Switzerland).

The reagents were used in our experiments at concentrations ranging from 10 μM to 500 μM. For the vehicle control, the DMSO or methanol concentration was selected to correspond to the highest measured inhibitor concentration (500 μM). For IBMX and Ibudilast, this corresponds to a DMSO concentration of 0.5 %, and for Sildenafil to 1.67 % methanol. In the "0 μM" condition corresponding volume of PBS was added as a substitute for the volume used for the inhibitor solution or the DMSO/methanol vehicle.

### 2.2 Isolation of human platelets

Healthy donors gave their written informed consent to the collection of blood and confirmed that they had not taken any medication for at least 10 days that could interfere with platelet function. The procedures had local ethics committee approval (141/2018B02, University Hospital Tübingen, Tübingen, Germany).

Donor blood was collected in a citrate phosphate dextrose adenine (CPDA) monovette (Sarstedt, Nümbrecht, Germany) for flow cytometry and ex vivo thrombus formation assays or a citrate monovette (Sarstedt, Nümbrecht, Germany) for aggregometry experiments. Preparation of platelet-rich-plasma (PRP) was performed as described [15]. In short, the anticoagulated whole blood was subjected to centrifugation at 209×*g* for 20 min at room temperature. The resulting platelet rich plasma (PRP) was subsequently transferred to a separate tube. Thereafter, the whole blood was subjected to a second round of centrifugation at 826×*g* for 10 min at room temperature. The resultant platelet poor plasma (PPP) was transferred to a separate tube. The platelet count of PRP and PPP was determined with a SYSMEX hematology analyzer (Sysmex Cooperation, Kobe, Japan) and the PRP count adjusted to 200,000 platelets/μl by dilution with PPP.

### 2.3 Flow cytometry

Dual color platelet flow cytometry was performed as described [15,16]. PRP (1 × 10^6^ platelets per sample) was preincubated with PDE inhibitors for 10 min at room temperature before stimulation with different agonists (CRP-A, ADP or TRAP6). Subsequently, the fluorophore-labeled antibodies anti-CD62P-PE (Beckman Coulter, Brea, CA, USA) and PAC-1-FITC (BD Biosciences, Franklin Lakes, NJ, USA) were added. After 30 min of incubation at room temperature without stirring, the 50 μl samples were fixed with 250 μl 0.5 % paraformaldehyde. Measurements were performed immediately using a FACS-Calibur flow cytometer (BD Biosciences, Franklin Lakes, NJ, USA). Data were collected by CellQuest Pro software and analyzed using FlowJo software (version 10.8.0; BD Biosciences, Franklin Lakes, NJ, USA).

### 2.4 Light transmission aggregometry

Platelet aggregation was measured by light transmission aggregometry using a standard protocol as described previously [17]. PRP was preincubated with PDE inhibitors for 10 min at 37 °C. Afterwards, aggregation was induced by addition of platelet stimulating agonists (CRP-A, ADP or TRAP6) in the indicated concentrations. Aggregation was measured for 5 min at 1,000 rpm and 37° using a light transmission aggregometer (CHRONO-LOG Aggregometer 490-X, Chrono-Lop Corp., Havertown, PA, USA). Aggregation data were collected using Aggrolink8 software (Chrono-Lop Corp., Havertown, PA, USA).

### *2.5* *Ex vivo* thrombus formation

Human whole blood was collected in citrate-phosphate-dextrose-adenine (CPDA) and diluted 5:1 with PBS + Ca^2+^ resulting in a final calcium concentration of 940 ng/ml. Samples were incubated with PDE inhibitors and stained with the fluorochrome 3,3′-dihexyloxacarbocyanine iodide (DiOC_6_; 1 μM; Sigma Aldrich Co., St. Luis, MO, USA) for 10 min at room temperature. Afterwards, 1 ml per sample was perfused over a collagen-coated surface (100 μg/ml; Takeda Pharmaceutical Company, Tokyo, Japan) in a transparent flow chamber (Maastricht Instruments B. V., Maastricht, The Netherlands) at a shear rate of 1,000 s^−1^. Perfusion was recorded on video for 2 min (1 s/frame, Nikon Eclipse Ti2-A, 20*x* objective). After rinsing the chamber with 1 ml PBS, five representative areas were imaged with a fluorescence microscope (Nikon Eclipse Ti2-A, 20*x* objective) and analyzed by NIS-Elements AR software (Version 5.21.00, Nikon Instruments Europe BV, Amsterdam, The Netherlands) [18].

### 2.6 Western blot analysis

Western blot analysis was done as described previously [19]. In short, isolated platelets were induced as indicated and incubated for 30 min at RT. The samples were mixed with RIPA lysis buffer and incubated for 10 min on ice. Protein concentrations were determined using a standard Bradford assay. 1:4 SDS loading buffer was added to the samples and 50 μg protein was loaded onto acrylamide gels (10 %) for electrophoretic separation. Standard wet blot techniques were applied for the protein transfer onto PVDF membranes. Membranes were blocked for 1 h with 5 % BSA in tris-tween buffered solution (TTBS), the primary antibody incubation was executed overnight at 4 °C in TTBS with 3 % BSA. After several wash steps the secondary antibody was incubated for 60 min at RT in TTBS with 3 % BSA. Subsequently the blots were washed and dried out before they were scanned using a LICOR scanner and analyzed using Image J.

### 2.7 Statistics and graphical presentation

GraphPad Prism software (GraphPad Software, Inc., La Jolla, CA, USA) was used to create graphs and test data for significance. Biorender.com was used to create the graphical abstract.

## 3 Results

### 3.1 Differential effects of PDE inhibitors on platelet fibrinogen receptor activation and degranulation of α-granula

**Fig. 1:**
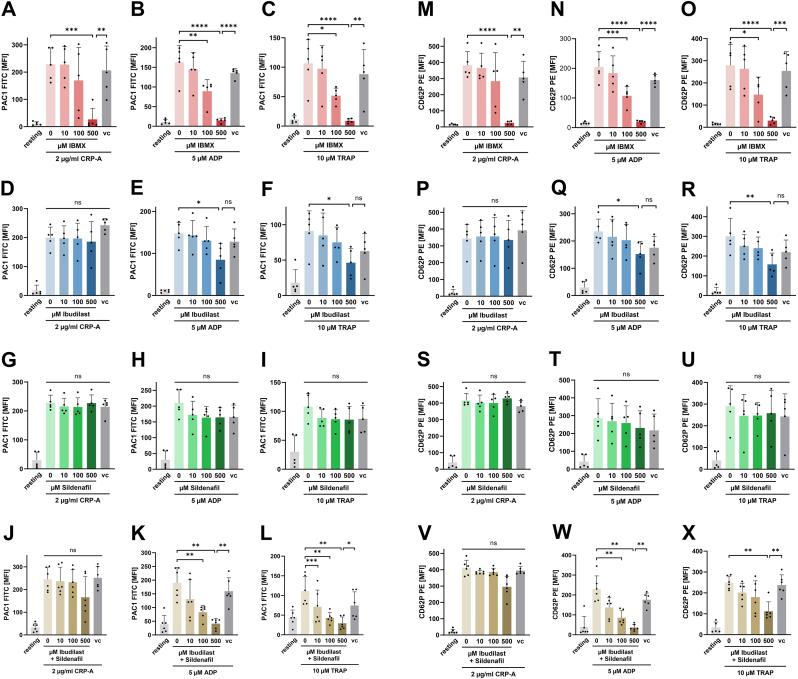
Effect of PDE inhibitors on platelet fibrinogen receptor activation and α-degranulation. **A-L** Statistical analysis of flow cytometry measurements of activated integrin IIb/IIIa (antibody clone PAC-1) surface expression. Human PRP was pre-treated with **A-C** IBMX, **D-F** Ibudilast, **G-I** Sildenafil or **J-L** combination of Ibudilast and Sildenafil and then stimulated with CRP-A, ADP or TRAP6 as indicated. vc vehicle control. Plotted: Mean ± SD, n ≥ 5, ordinary one-way ANOVA; ns not significant, ∗p < 0.05, ∗∗p < 0.01, ∗∗∗p < 0.001, ∗∗∗∗p < 0.0001. **M-X** Statistical analysis of flow cytometry measurements of CD62P surface expression. Human PRP was pretreated with **M-O** IBMX, **P–R** Ibudilast, **S–U** Sildenafil or **V-X** combination of Ibudilast and Sildenafil and then stimulated with CRP-A, ADP or TRAP6 as indicated. vc vehicle control. Plotted: Mean ± SD, n ≥ 4, ordinary one-way ANOVA; ns not significant, ∗p < 0.05, ∗∗p < 0.01, ∗∗∗p < 0.001, ∗∗∗∗p < 0.0001.

To evaluate the effect of the different selective and non-selective PDE inhibitors on platelet activation, isolated human platelets were stimulated with 2 μg/ml CRP-A, 5 μM ADP or 10 μM TRAP6 in the presence of various concentrations of PDE inhibitors. Activation of fibrinogen receptor GPIIb-IIIa was assessed by using the activation-specific monoclonal antibody PAC-1 and release of α-granules was characterized via P-selectin surface expression (binding of labeled anti-CD62P antibody) using flow cytometry (Fig. 1).

We found that the nonspecific PDE inhibitor IBMX inhibits activation of GPIIb-IIIa in a concentration-dependent manner irrespectively of the type of agonist used to induce platelet activation (Fig. 1A–C). In a concentration of 500 μM, IBMX reduced activation of GPIIb-IIIa to the level of untreated, resting platelets in response to all used agonists (CRP-A: p < 0.001; ADP: p < 0.0001; TRAP6: p < 0.0001). In a medium concentration of 100 μM, IBMX reduced GPIIb-IIIa activation induced by ADP (p < 0.01) and TRAP6 (p < 0.05) but not CRP. In contrast, the non-selective inhibitor Ibudilast that preferentially modulates PDE-3, attenuates PAC-1 binding induced by ADP and TRAP (p < 0.05) but not CRP-induced PAC-1 binding (Fig. 1D–F). The PDE5-specific inhibitor Sildenafil did not show a significant effect on GPIIb-IIIa expression on activated platelets (Fig. 1G–I). The combination of Ibudilast and Sildenafil was able to reduce ADP- and TRAP-induced integrin activation to a greater extent than Ibudilast and Sildenafil alone but had no effect on CRP-mediated platelet activation (Fig. 1J–L).

Similar results were found for the release of α-granules (P-selectin surface expression). In a concentration of 500 μM, IBMX strongly inhibits surface expression of P-selectin in response to CRP-A (p < 0.0001), ADP (p < 0.0001) and TRAP6 (p < 0.0001), 100 μM IMBX still had a significant inhibitory effect. (Fig. 1M and O). High Ibudilast concentrations particularly decrease P-selectin expression on ADP- (p < 0.05) and TRAP-stimulated (p < 0.01) but not on CRP-activated platelets (Fig. 1P–R). No substantially decrease of α-degranulation was noted in the presence of Sildenafil (Fig. 1S–U). The combination of Ibudilast and Sildenafil reduced ADP- and TRAP-mediated P-selectin expression to a greater extent than Ibudilast and Sildenafil alone and did not affect CRP-mediated platelet degranulation (Fig. 1V–X).

### 3.2 Effect of PDE inhibitors on platelet aggregation

**Fig. 2:**
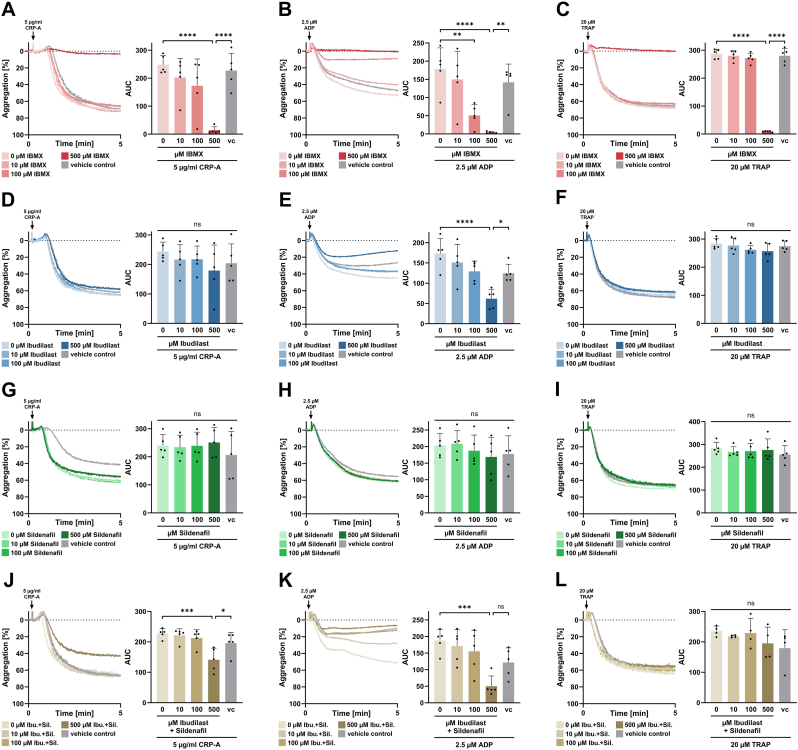
Effect of PDE inhibitors on platelet aggregation. **A-L** Representative curves and statistical analysis of light transmission aggregometry measurements. CRP-A-, ADP- or TRAP6-induced aggregation at 37 °C in human PRP after pre-treatment with **A-C** IBMX, **D-F** Ibudilast, **G-I** Sildenafil or J-L combination of Ibudilast und Sildenafil as indicated. vc vehicle control. Plotted: Mean ± SD; n ≥ 4, ordinary one-way ANOVA; ∗p < 0.05; ∗∗p < 0.01; ∗∗∗∗p < 0.0001.

To assess the differential effects of PDE inhibitors on platelet aggregation, PRP was incubated with PDE inhibitors and agonist-induced platelet aggregation was analyzed by light transmission aggregometry. As described above for platelet activation and α-degranulation, the non-selective PDE-inhibitor IBMX substantially inhibits platelet aggregation in a concentration-dependent manner irrespectively of the type of agonist used to induce platelet activation (Fig. 2A–C). Ibudilast specifically decreases ADP-induced platelet aggregation (p < 0.0001), but not CRP- or TRAP-mediated aggregation (Fig. 2D–F). No significant effect on platelet aggregation was noted in the presence of Sildenafil (Fig. 2G–I). The combination of Ibudilast and Sildenafil inhibited CRP-induced platelet aggregation, which the inhibitors alone were unable to do, and had a similar effect on ADP-mediated aggregation as observed with Ibudilast alone (Fig. 2J–L).

### 3.3 Platelet-dependent thrombus formation on immobilized collagen under flow is dependent on PDE

**Fig. 3:**
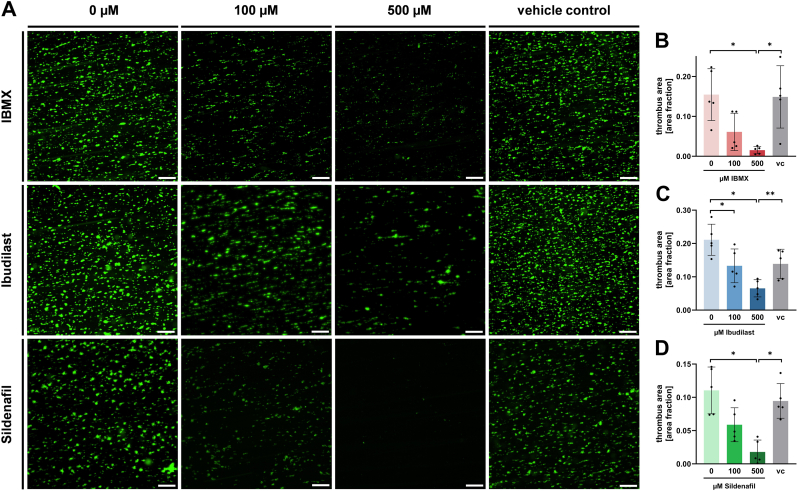
Effect of PDE inhibitors on thrombus formation on immobilized collagen under flow. **A** Representative fluorescence microscopy images of thrombi stained with DiOC_6_ (scale: 100 μM) after human whole blood was perfused over a collagen-coated surface at a shear rate of 1.000 s^−1^. **B-D** Statistical analysis of thrombus area (area fraction) after pretreatment of whole blood with **B** IBMX, **C** Ibudilast or **D** Sildenafil as indicated. vc vehicle control. Plotted: Mean ± SD, n = 5, Repeated measures one-way ANOVA; ns not significant, ∗p < 0.05, ∗∗p < 0.01.

We found that in the presence of all tested PDE inhibitors, platelet-dependent thrombus formation was substantially reduced (p < 0.05) (Fig. 3B–D).

### 3.4 Effect of PDE inhibitors on VASP phosphorylation

**Fig. 4:**
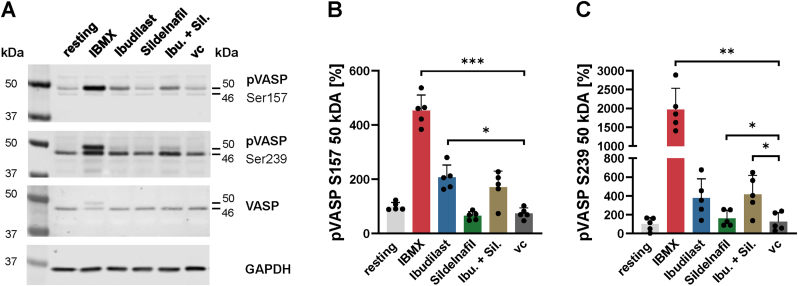
**A** Representative western blots images of IBMX, Ibudilast and Sildelnafil treated platelet samples, first panel pVASP S157, second panel pVASP S239, third panel VASP and fourth panel GAPDH loading control. **B/C** Densitometric analysis of the 50 kDa signal of pVASP S157 and pVASP S329 under stimulation **B** pVASP S157 and **C** pVASP S329. vc vehicle control. Plotted: Mean ± SD; n = 5, repeated measure one-way ANOVA; ∗p < 0.05, ∗∗p < 0.01, ∗∗∗p < 0.001.

We performed a western blot analysis of the VASP phosphorylation by the PDE inhibitors IBMX, Ibudilast and Sildelnafil each applied at 500 μM. Our western blot results demonstrate a robust phosphorylation dependent shift of the molecular weight of pVASP Ser157 from 46 kDa to 50 kDa under Ibudilast stimulation (Fig. 4A–C). This shift was weak in case of pVASP Ser239 detection. PKA preferentially phosphorylates at Ser157 followed by Ser239. Ser239 in contrast is a major PKG phosphorylation site. These results also demonstrate a preference of Ibudilast for PDE-3 inhibition (PKA activation) over PDE-5 inhibition (PKG activation).

IBMX in contrast induces a significant phosphorylation of Ser157 and Ser239 and corresponding shift in the molecular weight (Fig. 4A–C). Hence, IBMX strongly induce PKA and PKG mediated VASP phosphorylation as would be expected of a broad band PDE inhibitor which compasses PDE-2, -3 and -5.

Ibudilast is inducing VASP phosphorylation but to a smaller extend then IBMX, thus explaining the stronger inhibitory effects of IBMX compared to Ibudilast. Sildelnafil as PDE-5 inhibitor would be expected to strongly increase the PKG activity, but only minimal increase of PKG induced VASP phosphorylation was observed (Fig. 4A–C). This could explain why Sildelnafil does not show any substantial effects in PRP and the need for additional blood derived factors or shear stress to show the full inhibitory potential of Sildelnafil observed in the ex vivo thrombus formation.

Also, the combination of Ibudilast and Sildelnafil has no additive effect on VASP phosphorylation on either S157 or S239.

## 4 Discussion

The key results of this study are: (1) PDE inhibitors have differential effects on platelet fibrinogen receptor activation and degranulation of α-granules. Both fibrinogen receptor activation and degranulation of α-granules is primarily regulated through PDE-2 and PDE-3 but not PDE-5. (2) Inhibition of platelet aggregation requires comprehensive inhibition of various platelet PDE isoforms and depends on the type of agonist used to induce platelet activation. (3) In contrast, ex vivo platelet-dependent thrombus formation was significantly decreased by all tested PDE inhibitors including PDE-5 inhibitors. Our findings imply that all PDEs present in platelets are involved in regulation of platelet function. Thus, although chronic administration of PDE inhibitors targeting PDE-2, 3, and 5 are not associated with clinically significant bleeding events [11], the combination of PDE inhibitors with other antithrombotic drugs such as Aspirin, P_2_Y_12_ blockers or direct oral anticoagulants might have a significant impact on bleeding tendencies in humans.

PDE-inhibitors are used successfully for a long time to treat a variety of diseases including peripheral occlusive disease (e.g., Cilostazol), pulmonary arterial hypertension (e.g., Tadalafil), or erectile dysfunction (e.g., Sildenafil) [11]. It is well known that PDEs play a pivotal role in the modulation of platelet activity, acting as a regulator of intracellular cAMP and cGMP levels [10]. Since increased platelet activity is associated with numerous diseases, inhibitory drugs of PDE isoforms are of therapeutic interest [3]. Two of the three in the present study tested PDE inhibitors are in clinical use to treat primary pulmonary hypertension/erectile dysfunction (Sildenafil) [7,20] or multiple sclerosis or asthma (Ibudilast) [21]. However, the effect of both Sildenafil and Ibudilast on platelet function has been poorly elaborated so far [[22], [23], [24]].

To characterize the effect of PDE inhibition on platelet function, we evaluated three PDE inhibitors with distinct selectivity on various PDEs found in platelets. We found that a comprehensive inhibition of platelet function requires a nonselective PDE inhibitor (IBMX) that targets all PDEs expressed in platelets or the combination of several selective PDE inhibitors. Targeting only a single PDE (PDE-5, Sildenafil or PDE-3, Ibudilast) is not sufficient for a comprehensive attenuation of platelet function regarding platelet fibrinogen receptor activation, degranulation and aggregation. Since Sildenafil (PDE-5 inhibitor) preferentially modulates cGMP levels (no significant effects on degranulation or aggregation) and Ibudilast (PDE-3 inhibitor) primarily enhances cAMP levels (only partially effects on degranulation or aggregation) our data imply that platelet degranulation and aggregation is predominantly regulated by cAMP. The observations made in the functional assays are corroborated by the analysis of VASP phosphorylation. This analysis indicates that non-selective inhibition of PDEs by IBMX results in significant VASP phosphorylation, while the inhibition of PDE3 by Ibudilast leads to comparatively low phosphorylation. Furthermore, only minimal increase in phosphorylation can be observed under static conditions with the PDE5 inhibitor Sildenafil under the applied concentrations.

In contrast, when the PDE inhibitors were tested in a dynamic platelet adhesion assay under flow and high shear rates, we found that all tested PDE inhibitors including the PDE-5 inhibitor Sildenafil substantially reduced platelet-dependent thrombus formation. Thus, it is tempting to speculate that shear-dependent platelet functions require primarily or exclusively contribution of the cGMP/PDE-5 signaling pathway [6,25]. Thus, depending on the nature of the applied platelet function assay differential effects of PDE inhibitors are disclosed.

Previously, it has been shown that Sildenafil affects collagen-mediated platelet activity exclusively in the presence of nitric oxide (NO) [26]. Further, it was shown that shear stress during thrombus growth activates the NO-cGMP pathway which modulates as an auto-regulatory brake platelet-dependent thrombus formation [25]. Thus, our results indicate that suppression of thrombus formation on immobilized collagen under flow requires at least the inhibition of the cGMP-hydrolyzing isoforms PDE-2 and PDE-5. Given that the cAMP-hydrolyzing PDE-3 is inhibited by cGMP, the inhibition of PDE-2, PDE-5 or both results in elevating cAMP levels as well and thus attenuates flow-induced platelet thrombosis. However, in our study the sole inhibition of PDE-3 by the nonspecific inhibitor Ibudilast is insufficient to affect CRP-mediated platelet activation and aggregation. This finding is contradicted by the results of a study which demonstrated that the selective PDE-3 inhibitor Cilostazol is able to reduce collagen-induced aggregation and P-selectin expression [27]. However, it is important to note that the IC_50_ value for PDE-3 of Cilostazol (0,2 μM) [28] is significantly lower than that of Ibudilast (31 μM) [24].

In contrast to collagen receptor (GPVI) mediated platelet stimulation, platelet activation by ADP via P_2_Y_12_ showed a greater sensitivity to PDE inhibitors, responding not only to IBMX but also to Ibudilast. IBMX and Ibudilast attenuated ADP-mediated GPIIb-IIIa activation, P-selectin expression, and platelet aggregation. Similar results were observed when platelets were stimulated with TRAP6 via the thrombin receptor PAR1. These results indicate, that the sole inhibition of the cAMP-hydrolyzing PDE-3 is sufficient to reduce ADP- and partly TRAP-mediated platelet activation but not collagen/GPVI-dependent platelet function.

In conclusion, the findings of the present study imply that the three PDE isoforms expressed in platelets are involved in regulating platelet function dependent on distinct activation pathways. The results indicate that chronic modulation of PDEs affects hemostasis and thrombosis in humans treated with PDE inhibitors which may enhance the risk of bleeding especially when administered in combination with antithrombotic drugs.

## CRediT authorship contribution statement

**Ravi Hochuli:** Writing – original draft, Visualization, Validation, Methodology, Investigation, Formal analysis. **Valerie Dicenta:** Writing – review & editing, Visualization, Validation, Methodology. **Zoi Laspa:** Writing – review & editing, Visualization, Validation, Methodology. **Manuel Sigle:** Writing – review & editing, Validation, Investigation. **Tobias Harm:** Writing – review & editing, Validation, Investigation. **Tatsiana Castor:** Writing – review & editing, Validation, Project administration. **Anne-Katrin Rohlfing:** Writing – review & editing, Visualization, Validation, Supervision, Conceptualization. **Meinrad Paul Gawaz:** Writing – review & editing, Writing – original draft, Validation, Supervision, Resources, Data curation, Conceptualization.

## Data availability statement

For original data please contact the corresponding author.

## Declaration of generative AI in scientific writing

No generative AI or AI-assisted technologies were used in the preparation of this manuscript.

## Funding

This research was supported by the 10.13039/501100001659Deutsche Forschungsgemeinschaft (10.13039/501100001659DFG, 10.13039/501100001659German Research Foundation) – Project number 335549539 – GRK2381. Ravi Hochuli was supported by a research grant of the 10.13039/501100010578German Cardiac Society (10.13039/501100010578DGK).

## Declaration of competing interest

All authors of the manuscript have no conflict of interest to declare.
